# Hospitals of the North-West

**Published:** 1891-11-21

**Authors:** 


### Hospitals of the North-West.

By our Special Commissioner.

### II.—VANCOUVER.

The medical institutions of Vancouver consist of a General Hospital, which is the property of the Town Council; St. Luke's Home, under the management of Sister Frances; and the Hospital for Women, a private venture in course of erection.

The General Hospital is entered by a staircase many feet high and very steep, up which it would be difficult, if not impossible, to carry an accident case without adding to its injuries. The building consists of one large ward, crowded with beds, without adequate ventilation or light, the atmosphere of which would be detrimental to a healthy person and, much more so, to a sick man. It would, in our judgment, be criminal to attempt to treat an open wound in this ward under present conditions. There are two smaller wards on the first floor containing a few beds, one of which is sometimes devoted to women, the other to typhoid and surgical cases. Every kind of disease, except infectious cases, seems to be admitted.

No trained nurse or qualified Matron is attached to the hospital. Serious complaints are made of the food, and, at the present time, the nursing is done by two devoted ladies —Miss Woodward and Miss Kenrick—who share the same bedroom, and have no sitting room of their own. They have, by hard work and length of service, acquired a fair knowledge of nursing, and it is highly creditable to them that they should have consented to work in this hospital under the present conditions, which are inimical to their health, opposed to their comfort, and well calculated to make them despair of their patients, owing to defective construction and insanitary arrangements.

The present buildings will be reconstructed. The upper floor approached by a separate entrance and staircase, will contain accommodation for the nursing staff and officers, whilst the ground floor will be turned into an isolation ward, where cases of infectious disease may be treated from time to time as occasion arises. It is proposed, owing to the small amount of money at present available, to proceed immediately with the hospital block proper, which will contain four wards, having eight beds each, and, at the same time, to reconstruct the old building.

The City Engineer, who is the architect of the new buildings, has shown a knowledge of the principles which govern hospital construction, and there is reason to believe that the new wards, when erected, will be complete and satisfactory. Each ward will have rounded corners, a central fireplace, and Louvre windows, and each bed will be so placed as to have a window on each side of it, care being taken to provide adequate cubic and superficial space. The lavatories and bath-rooms will be quite distinct from the wards, and will be entered from the verandah, which will be placed all round the building on each floor. It is intended that the drains shall be disconnected from the sewers by a manhole and syphon trap, the waste pipes of the baths and lavatories shall terminate outside and above an open grating, and that the soil pipes shall be carried through an open manhole so that a free current of fresh air may always circulate through these pipes. The Mayor and Board of Health agreed at an informal conference with the writer to organise a nursing staff, to take steps to secure a lady who possessed a certificate of training, and who, was also an experienced, able, and wise administrator. The nurses are to have a separate sitting room, and each member of the staff will be supplied with a separate bedroom for herself.

Since the above was written, we have had an interview with the Mayor and Board of Health, and, at their request, have gone through the plans of the proposed extension of the Vancouver Hospital, and have made certain suggestions and additions; and, after a conference, the authorities have pledged themselves to put the hospital in a thorough state of efficiency without delay. Under these circumstances, it is unnecessary to describe this building in detail, as the Corporation, on the advice of the Health Committee, have decided to erect a new hospital. The new buildings will consist of three blocks, devoted respectively to the (1) administration; (2) laundry and kitchen; and (3) the wards and necessary offices only.

If these points are attended to, and the whole of the suggested arrangements be carried out as agreed by the Mayor and Board of Health, then the hospital at Vancouver will be a credit to the town. We shall watch the work as it proceeds, and shall hope to be able to report before long that the Municipal Hospital at Vancouver is so organised and constructed as to compare favourably with any similar institution in the British Colonies.

In this connection we may mention that Victoria has erected a new hospital, which we had no opportunity to visit, but which is reported to be replete with every modern appliance. It consists of upwards of 50 beds, and so far as construction is concerned, at any rate, the inhabitants of Victoria have shown much more wisdom than those at Vancouver. Unfortunately, the domestic management of the hospital, we were informed, is not, at present, in the hands of a trained and certificated nurse, and, until the administration has been reorganised and placed upon a modern basis in these respects, it is not possible to hope that the institution will do the maximum of good work in the best possible manner.

St. Luke's Home.

St. Luke's Home, Vancouver, of which a full report has been given in the "Nursing Mirror" more than once, reflects great credit upon all concerned. It consists of a good modern house, with bath-rooms, etc., containing a number of beds, where private cases are received, which pay from ten to fifteen dollars a week each. This charge includes everything except medical attendance, and for the higher fee-patients have the advantage of being treated in a private room. The ten-dollar patients are placed in a ward containing four or five beds. Having regard to the condition of the hospital in Vancouver, and to the fact that nowhere, except at St. Luke's Home, can a patient who desires to pay be adequately provided for, we would urge the residents to come forward and interest themselves in St. Luke's Home, and to take care, as a matter of insurance, for the sake of themselves and their families, that adequate funds are forthcoming to enable Sister Frances not only to maintain the home in its present state of efficiency, but to extend it from time to time as may be found necessary or desirable.

